# Editorial for the Special Issue “Gut Dysbiosis: Molecular Mechanisms and Therapies 2.0”

**DOI:** 10.3390/biomedicines12102186

**Published:** 2024-09-26

**Authors:** Carmine Stolfi, Federica Laudisi

**Affiliations:** Department of Systems Medicine, University of Rome “Tor Vergata”, 00133 Rome, Italy; carmine.stolfi@uniroma2.it

Gut homeostasis depends on maintaining a fine equilibrium between the intestinal epithelial barrier, the microbiota, and the host’s immune system. Effective modulation by these three actors is mandatory to protect against pathogens and guarantee the absorption of water and nutrients, thus ensuring intestine health [1,2,3]. Unfortunately, several factors can undermine this balance, leading to the development of a variety of inflammatory disorders [4,5,6]. This Special Issue comprises three original studies and three review articles written by leading international experts in the field, focusing on the impacts of environmental factors on intestinal homeostasis and potential therapeutic interventions available to treat gut dysbiosis.

The world that we live in includes a wide range of stimuli that put strain on gut homeostasis. This is the case for dietary factors, which are known to greatly shape the intestinal microbiota community and immune response [7]. For example, the acquisition of Westernized dietary habits, characterized by high sugar, saturated fatty acid and salt intake, as well as low amounts of fiber and fruit, was found to correlate with a huge increase in the frequency of patients affected by chronic intestinal inflammatory diseases [8,9]. In this context, it is important to consider that the Western diet often encompasses consuming ultra-processed food (UPF), resulting from several industrial steps with significant use of food additives and specific cooking methods. Stolfi and co-workers (contribution 1) reviewed the impact of the Western diet and UPF on the composition of the gut microbiota, the immune response and the integrity of the intestinal barrier. The intake of UPF alters goblet cell function and, ultimately, mucus production, thus affecting the first line of defense against pathogens and dietary antigens. Furthermore, a change in microbial composition after UPF intake can stimulate the growth of mucus-degrading bacteria, further compromising the thickness of the mucus layer.

As mentioned previously, UPF undergoes several industrial processes and specific cooking methods (e.g., dry heating, frying) that promote the non-enzymatic reaction of proteins, aminoglycosides, and amino-terminal lipids with reducing sugars, followed by a sequence of Amadori rearrangements and oxidative modifications, leading to the generation of advanced glycation end products (AGEs) [10]. These compounds can interact with the receptor for advanced glycation end products (RAGEs) and trigger several pathological mechanisms (e.g., the production of pro-inflammatory cytokines, oxidative stress, the alteration of the composition of the gut microbiota). In fact, growing evidence correlates the accumulation of AGE with the onset and progression of different diseases, such as diabetes, atherosclerosis, and neurological disorders (including Alzheimer’s disease, the secondary effects of traumatic brain injury, and amyotrophic lateral sclerosis) [11,12,13]. Reddy et al. (contribution 2) provide a comprehensive overview of the effects of AGE-RAGE interactions in neurological pathologies and discuss the potential therapeutic strategies involving the use of RAGE antagonists (i.e., Azeliragon and FPS-ZM1) in clinical practice. The authors report that the AGE-RAGE interaction may be impaired by the use of small-molecule-based or cytoplasmic tail-based RAGE antagonists, which successfully reduce the oxidative stress and plasma concentrations of inflammatory cytokines, and re-establish commensal community.

Despite their potential detrimental role in promoting gut dysbiosis, dietary components and/or microorganisms may represent an important tool for maintaining gut homeostasis and intestinal barrier function in pathological conditions.

Patel et al. (contribution 3) investigated the effects of a synbiotic treatment, in which a prebiotic compound helps the probiotic organism to survive and grow within the intestinal tract, on the development and progression of alcoholic liver disease (ALD), a pathologic condition encompassing the liver manifestations of alcohol overconsumption and influenced by the gut–liver axis [14]. In particular, by examining the integrity of the colon barrier, oxidative stress, and inflammation in human epithelial colon cells (Caco-2) and alcohol-induced colon injury in male Wistar rats, the authors suggest that a combination of aged garlic extract and *Lactobacillus rhamnosus* MTCC1423 may help with the management of ALD and, theoretically, chronic intestinal inflammation, such as that occurring in patients with inflammatory bowel diseases (IBDs). However, it should be noted that some synbiotics were tested in patients with Crohn’s disease (CD) and ulcerative colitis (UC), with contrasting results [15,16], suggesting that greater efforts need to be made in this direction.

This need is even greater in pediatric IBD, a chronic inflammatory intestinal disease that affects both children and adolescents and whose symptoms can significantly affect the growth, development, and quality of life of a child, making early diagnosis and effective management crucial [17]. The study by Dovrolis and colleagues (contribution 4) focuses on treatment-naïve pediatric IBD patients and their immediate families to identify the role of the microbiome in the onset of the disease. By performing 16S ribosomal RNA gene sequencing and bioinformatic analysis of fecal samples collected from seven drug-naïve CD patients and two drug-naïve UC patients, as well as twenty-four healthy siblings/parents, the authors identified patterns of dysbiosis and hallmark microbial taxa among patients who shared ethnic, habitual, and dietary traits with themselves and their families, suggesting the potential roles of maternal factors in the establishment and modulation of the early life microbiome, which may have implications for our understanding of the etiology and progression of IBD.

Fecal microbiota transplantation (FMT), a therapeutic strategy based on the use of fecal enemas from healthy donors to improve the diversity and abundance of the host’s gut microbiota, can be employed to treat patients with gut microbial imbalance [18].

While this approach is particularly successful for treating *Clostridium difficile* infection [19], concerns related to safety and efficacy persist for the treatment of other pathological conditions (for more information, see https://www.fda.gov/safety/medical-product-safety-information/fecal-microbiota-transplantation-safety-communication-risk-serious-adverse-reactions-due, accessed on 26 August 2024). Several studies have been performed in patients with UC, which had good results in terms of clinical and endoscopic remission, while long-term effects, as well as safety issues, are still debated [20]. Furthermore, the use of FMT has also been investigated in order to reduce the adverse effects of colitis in patients receiving immune checkpoint inhibitors [21]. Other applications include the treatment of obesity and metabolic syndrome, as well as extraintestinal pathologies such as neurological diseases (e.g., autistic spectrum disorders, Parkinson’s disease) and hematopoietic malignancies [22,23,24]. Concerning this latter case, Li and colleagues (contribution 5) reported interesting preliminary observations in patients who received autologous FMT after conditioning chemotherapy and haematopoietic stem cell transplantation (HSCT) to prevent adverse effects related to the alteration of the host’s microbiota and donor-to-receipt disease transmission. Even if this is an exploratory study with evident limitations due to the logistical challenges of autologous stool collection and the very small sample size, it allows us to collect stool from patients during the acute phase instead of later stages, such as after first-line therapy and before HSCT, as previously reported, to prevent alteration in microbial richness and survival.

Although the above-mentioned therapeutic approaches provided interesting results and are constantly improving, they rely on the use of in vitro and in vivo models that only partially highlight the mechanisms involved in host–microbial interactions. In this context, the advent of gut-on-chip technology represents the generation of a valuable tool to improve our knowledge in this respect, thanks to the ability to reproduce a dynamic system such as the intestinal mucosa, which includes the host barrier, immune cells, and microbes [25,26]. The comprehensive review article by Morelli et al. (contribution 6) summarizes the key parameters of gut-on-chip models to study host–microbial interactions. The authors also highlight the advantages and disadvantages of each model and discuss the challenges that the field must address, such as standardization and scalability, to advance our understanding of disease mechanisms and improve the efficacy of patient-tailored treatments.

In conclusion, understanding the microbiota–gut–organ axis has opened the door to a better appreciation of different disease pathologies and offered opportunities to study therapeutics acting through the regulation of the microbiome. However, further research is needed in this area to clarify pathways, mechanisms, and benefits to human health. We hope that the articles and reviews in this Special Issue meet the expectations of readers in the field and further promote studies by the scientific community on the mechanisms that trigger and/or maintain gut dysbiosis, ultimately leading to more personalized and effective treatments.

## Data Availability

Not applicable.
